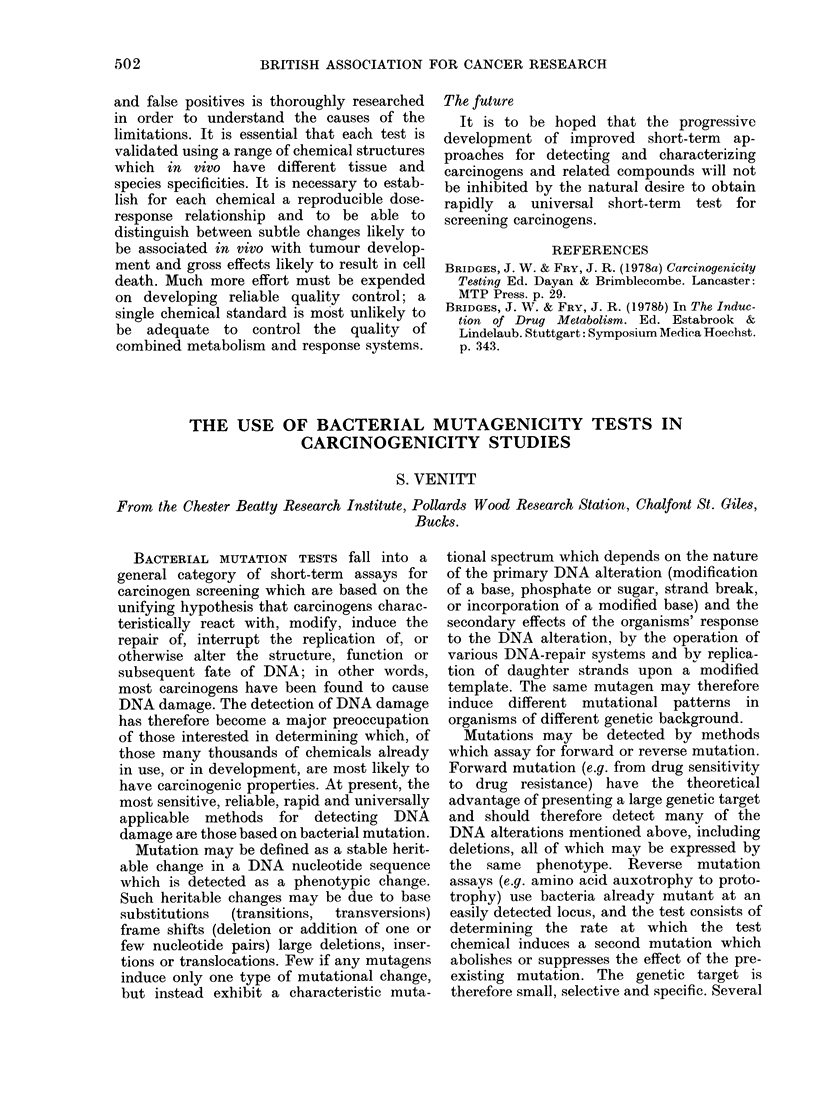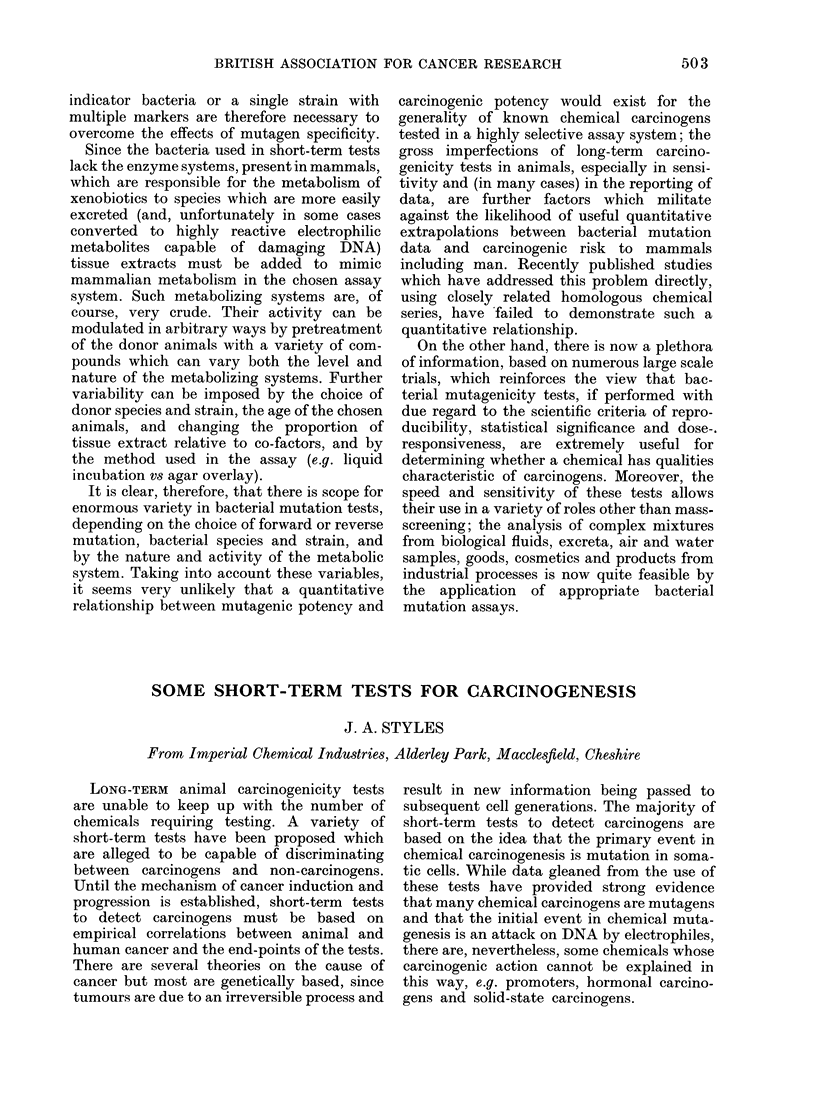# The use of bacterial mutagenicity tests in carcinogenicity studies.

**DOI:** 10.1038/bjc.1980.82

**Published:** 1980-03

**Authors:** S. Venitt


### THE USE OF BACTERIAL MUTAGENICITY TESTS IN CARCINOGENICITY STUDIES

S. VENITT

*From the Chester Beatty Research Institute, Pollards Wood Research Station, Chalfont St. Giles, Bucks.*

BACTERIAL MUTATION TESTS fall into a general category of short-term assays for carcinogen screening which are based on the unifying hypothesis that carcinogens characteristically react with, modify, induce the repair of, interrupt the replication of, or otherwise alter the structure, function or subsequent fate of DNA; in other words, most carcinogens have been found to cause DNA damage. The detection of DNA damage has therefore become a major preoccupation of those interested in determining which, of those many thousands of chemicals already in use, or in development, are most likely to have carcinogenic properties. At present, the most sensitive, reliable, rapid and universally applicable methods for detecting DNA damage are those based on bacterial mutation.

Mutation may be defined as a stable heritable change in a DNA nucleotide sequence which is detected as a phenotypic change. Such heritable changes may be due to base substitutions (transitions, transversions) frame shifts (deletion or addition of one or few nucleotide pairs) large deletions, insertions or translocations. Few if any mutagens induce only one type of mutational change, but instead exhibit a characteristic mutational spectrum which depends on the nature of the primary DNA alteration (modification of a base, phosphate or sugar, strand break, or incorporation of a modified base) and the secondary effects of the organisms' response to the DNA alteration, by the operation of various DNA-repair systems and by replication of daughter strands upon a modified template. The same mutagen may therefore induce different mutational patterns in organisms of different genetic background.

Mutations may be detected by methods which assay for forward or reverse mutation. Forward mutation (*e.g.* from drug sensitivity to drug resistance) have the theoretical advantage of presenting a large genetic target and should therefore detect many of the DNA alterations mentioned above, including deletions, all of which may be expressed by the same phenotype. Reverse mutation assays (*e.g.* amino acid auxotrophy to prototrophy) use bacteria already mutant at an easily detected locus, and the test consists of determining the rate at which the test chemical induces a second mutation which abolishes or suppresses the effect of the pre-existing mutation. The genetic target is therefore small, selective and specific. Several indicator bacteria or a single strain with multiple markers are therefore necessary to overcome the effects of mutagen specificity.

Since the bacteria used in short-term tests lack the enzyme systems, present in mammals, which are responsible for the metabolism of xenobiotics to species which are more easily excreted (and, unfortunately in some cases converted to highly reactive electrophilic metabolites capable of damaging DNA) tissue extracts must be added to mimic mammalian metabolism in the chosen assay system. Such metabolizing systems are, of course, very crude. Their activity can be modulated in arbitrary ways by pretreatment of the donor animals with a variety of compounds which can vary both the level and nature of the metabolizing systems. Further variability can be imposed by the choice of donor species and strain, the age of the chosen animals, and changing the proportion of tissue extract relative to co-factors, and by the method used in the assay (*e.g.* liquid incubation *vs* agar overlay).

It is clear, therefore, that there is scope for enormous variety in bacterial mutation tests, depending on the choice of forward or reverse mutation, bacterial species and strain, and by the nature and activity of the metabolic system. Taking into account these variables, it seems very unlikely that a quantitative relationship between mutagenic potency and carcinogenic potency would exist for the generality of known chemical carcinogens tested in a highly selective assay system; the gross imperfections of long-term carcinogenicity tests in animals, especially in sensitivity and (in many cases) in the reporting of data, are further factors which militate against the likelihood of useful quantitative extrapolations between bacterial mutation data and carcinogenic risk to mammals including man. Recently published studies which have addressed this problem directly, using closely related homologous chemical series, have failed to demonstrate such a quantitative relationship.

On the other hand, there is now a plethora of information, based on numerous large scale trials, which reinforces the view that bacterial mutagenicity tests, if performed with due regard to the scientific criteria of reproducibility, statistical significance and dose-. responsiveness, are extremely useful for determining whether a chemical has qualities characteristic of carcinogens. Moreover, the speed and sensitivity of these tests allows their use in a variety of roles other than mass-screening; the analysis of complex mixtures from biological fluids, excreta, air and water samples, goods, cosmetics and products from industrial processes is now quite feasible by the application of appropriate bacterial mutation assays.